# The role of ferroptosis in breast cancer patients: a comprehensive analysis

**DOI:** 10.1038/s41420-021-00473-5

**Published:** 2021-05-04

**Authors:** Zeng-Hong Wu, Yun Tang, Hong Yu, Hua-Dong Li

**Affiliations:** 1 Department of Otorhinolaryngology, Union Hospital, Tongji Medical College, Huazhong University of Science and Technology, Wuhan, Hubei China; 2 Department of Infectious Diseases, Union Hospital, Tongji Medical College, Huazhong University of Science and Technology, 430022 Wuhan, China; 3 Department of Critical Care Medicine, Union Hospital, Tongji Medical College, Huazhong University of Science and Technology, 430022 Wuhan, China; 4 Department of Cardiovascular Surgery, Union Hospital, Tongji Medical College, Huazhong University of Science and Technology, 430022 Wuhan, China

**Keywords:** Immune cell death, Cancer genomics

## Abstract

Breast cancer (BC) affects the breast tissue and is the second most common cause of mortalities among women. Ferroptosis is an iron-dependent cell death mode that is characterized by intracellular accumulation of reactive oxygen species (ROS). We constructed a prognostic multigene signature based on ferroptosis-associated differentially expressed genes (DEGs). Moreover, we comprehensively analyzed the role of ferroptosis-associated miRNAs, lncRNAs, and immune responses. A total of 259 ferroptosis-related genes were extracted. KEGG function analysis of these genes revealed that they were mainly enriched in the HIF-1 signaling pathway, NOD-like receptor signaling pathway, central carbon metabolism in cancer, and PPAR signaling pathway. Fifteen differentially expressed genes (*ALOX15, ALOX15B, ANO6, BRD4, CISD1, DRD5, FLT3, G6PD, IFNG, NGB, NOS2, PROM2, SLC1A4, SLC38A1*, and *TP63*) were selected as independent prognostic factors for BC patients. Moreover, T cell functions, including the CCR score, immune checkpoint, cytolytic activity, HLA, inflammation promotion, para-inflammation, T cell co-stimulation, T cell co-inhibition, and type II INF responses were significantly different between the low-risk and high-risk groups of the TCGA cohort. Immune checkpoints between the two groups revealed that the expressions of *PDCD-1 (PD-1), CTLA4, LAG3, TNFSF4/14, TNFRSF4/8/9/14/18/25*, and *IDO1/2* among others were significantly different. A total of 1185 ferroptosis-related lncRNAs and 219 ferroptosis-related miRNAs were also included in this study. From the online database, we identified novel ferroptosis-related biomarkers for breast cancer prognosis. The findings of this study provide new insights into the development of new reliable and accurate cancer treatment options.

## Background

Breast cancer (BC) affects the breast tissue and easily metastasizes to the bones and lungs. Globally, BC has very high incidence rates^1^. It is the second most common cause of mortalities among women^2^, accounting for an estimated 24% of diagnosed cases and 15% of mortality cases among them^3^. However, due to the asymptomatic early stages, BC is often diagnosed in the advanced stages, leading to serious outcomes. Early diagnosis and intervention are important for favorable outcomes^4^. Based on genomic and transcriptomic sequences, BC can be allocated into five inherent molecular subtypes, including Luminal A/B, HER-2 enriched, Basal-like and Claudin-low^5^. Despite of the advances in diagnosis, surgery, chemotherapy and radiotherapy, BC is still an extremely malignant tumor with poor survival outcomes^6^. It has been found that BC has become tolerant to some antitumor drugs with apoptotic effects^7^. Thus, it is important to evaluate other potential forms of cell death to overcome chemotherapeutic resistance and to uncover new biomarkers and therapeutic targets for improving BC prognosis.

Studies on ferroptosis in tumors are on the ascendancy. Ferroptosis is an iron-dependent cell death mode that is characterized by intracellular accumulation of reactive oxygen species (ROS), which is distinct from apoptosis and autophagy in morphology^8^. The main mechanism of ferroptosis is that, under the action of divalent iron or ester oxygenase, it catalyzes the unsaturated fatty acids highly expressed on the cell membrane to cause liposome peroxidation, thereby inducing cell death. Dysregulation of iron metabolism can enhance the risk of cancer and facilitate tumor growth. Compared to normal cells, cancer cells are more dependent on iron, a phenomenon known as iron addiction^9^. Activation of ferroptosis pathways can overcome the drug resistance mechanisms associated with the existing chemotherapeutic agents, and ultimately provide new therapeutic options for treating cancer. Ferroptosis enhances metabolic dysfunctions that lead to the production of cytoplasmic and lipid ROS, independent of mitochondria while dependent on NADPH oxidases in certain cellular environments^10^. A recent study devised an Fe^2+^-based metal-organic skeleton, which transfers Fe^2+^ to cancer cells, thereby triggering Fenton reaction, producing excess ROS, and inducing ferroptosis in BC^11^. Moreover, ferroptosis resistance can be mediated by a prominin2-MVB-exosome-ferritin pathway in BC cells. Prominin2 is a pentaspanin protein involved in regulation of lipid dynamics^12^. However, very few studies have systematically evaluated ferroptosis-related molecular signatures to predict overall survival (OS) in BC patients. We established a prognostic multigene signature based on ferroptosis-related differentially expressed genes (DEGs) obtained from The Cancer Genome Atlas (TCGA). The signatures were validated in the Gene Expression Omnibus (GEO) cohort. In addition, the role of ferroptosis-related miRNAs, lncRNAs, and immune responses in BC were also evaluated.

## Results

### Enrichment analysis of ferroptosis-related genes

**Fig. 1:**
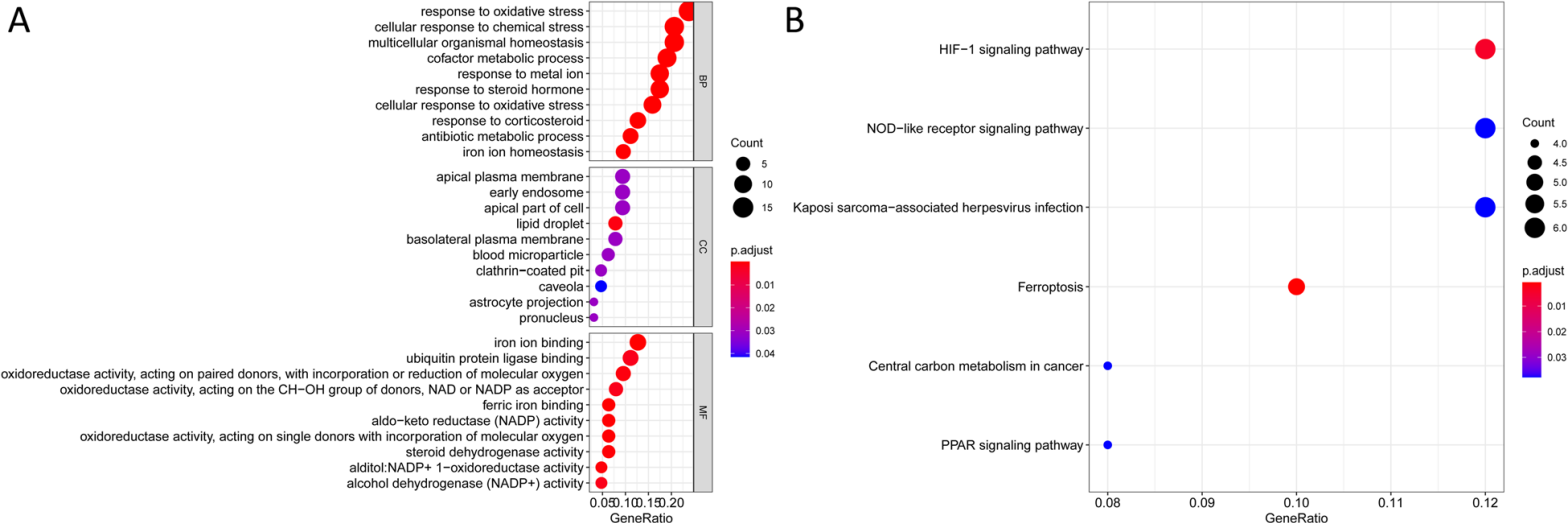
Gene ontology (GO) and Kyoto Encyclopedia of Genes and Genomes (KEGG) outcomes of ferroptosis-related genes through the R language “ggplot2” and “org.Hs.eg.db” package (adjust *p* value <0.05). The org.Hs.eg.db package implements the conversion between gene symbols and entrezIDs based on mget function. The *X*-axis represents the gene ratio, that is, the number of genes enriched in each GO/the total number of genes and the *Y*-axis represents the specific function or pathway that is enriched. The size of the circle represents the number of genes enriched on each GO, and the redder the color represents the higher the degree of enrichment on GO. **A** GO; **B** KEGG. (BP: biological processes, MF: molecular functions, CC: cellular components).

BP of target genes were enriched in response to oxidative stress, multicellular organismal homeostasis, cofactor metabolic processes, and response to metal ion activities among others; MF were mainly enriched in ferric iron binding, aldo-keto reductase (NADP) activity, and oxidoreductase activity among others; CC were mainly enriched in the regulation of epithelial cell proliferation, lipid droplet, apical plasma membrane, and astrocyte projection among others; KEGG were mainly enriched in ferroptosis, hypoxia-inducible factor (HIF)−1 signaling pathway, NOD-like receptor signaling pathway, central carbon metabolism in cancer, Kaposi sarcoma-associated herpesvirus infection, and PPAR signaling pathway (Fig. 1). A total of 65 (28 upregulated and 37 downregulated) DEGs were identified.

### Building the prognostic ferroptosis-based signature

**Fig. 2:**
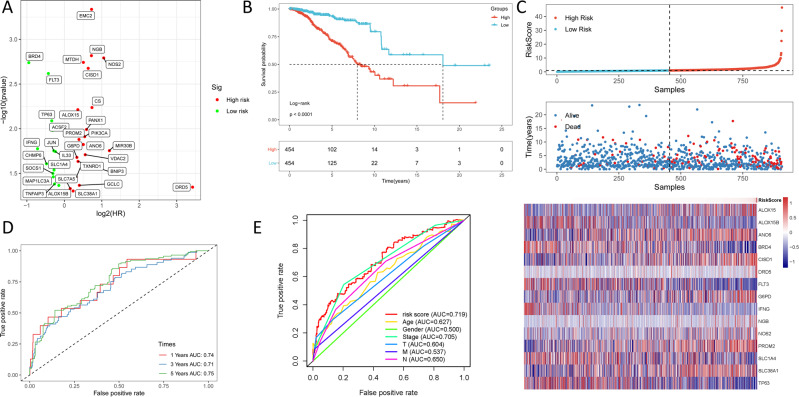
Evaluate the accuracy of the ferroptosis-related gene signature based on TCGA. **A** Univariate COX analysis identified 33 ferroptosis-related genes; red means upregulated and greed means downregulated genes; **B** Kaplan–Meier indicated that high-risk group (red) exhibited a poorer survival outcome when compared to the low-risk group (blue); **C** The top graph represents the classification of patients into high (red) and low (blue) risk groups based on risk scores, the middle graph represents as the patient’s risk score increased, the mortality rate also increased, the bottom heatmap represents the change of 15 ferroptosis-related genes as the risk score increases; **D** The AUC values of the patients’ 1, 3, 5-year survival rate; **E** the AUC of the signature was 0.719, exhibiting superior performance than the traditional clinicopathological features in predicting the prognosis.

To screen for prognostic ferroptosis, differentially expressed ferroptosis genes were evaluated in the univariate COX analysis. Thirty-three ferroptosis genes that were found to be significant in the univariate COX analysis were included in the multivariate COX analysis (Fig. 2A). As a result, 15 differently expressed genes (*ALOX15, ALOX15B, ANO6, BRD4, CISD1, DRD5, FLT3, G6PD, IFNG, NGB, NOS2, PROM2, SLC1A4, SLC38A1*, and *TP63*) were selected as independent prognostic factors for BC patients (Table S1). Thus, the formula for our model was: Risk Score = (0.177 × expression_ALOX15_) + (0.535 × expression_ANO6_) + (0.473 × expression_CISD1_) + (3.530 × expression_DRD5_) + (0.189 × expression_G6PD_) + (0.333 × expression_NGB_) + (0.672 × expression_NOS2_) + (0.260 × expression_PROM2_) + (0.159 × expression_SLC38A1_) − (0.109 × expression_ALOX15B_) − (0.705 × expression_BRD4_) − (0.160 × expression_FLT3_) − (0.541 × expression_IFNG_) − (0.289 × expression_SLC1A4_) − (0.242 × expression_TP63_). In addition, the risk score of each patient in our study cohort was calculated.

### Survival outcomes and multivariate examination

**Fig. 3:**
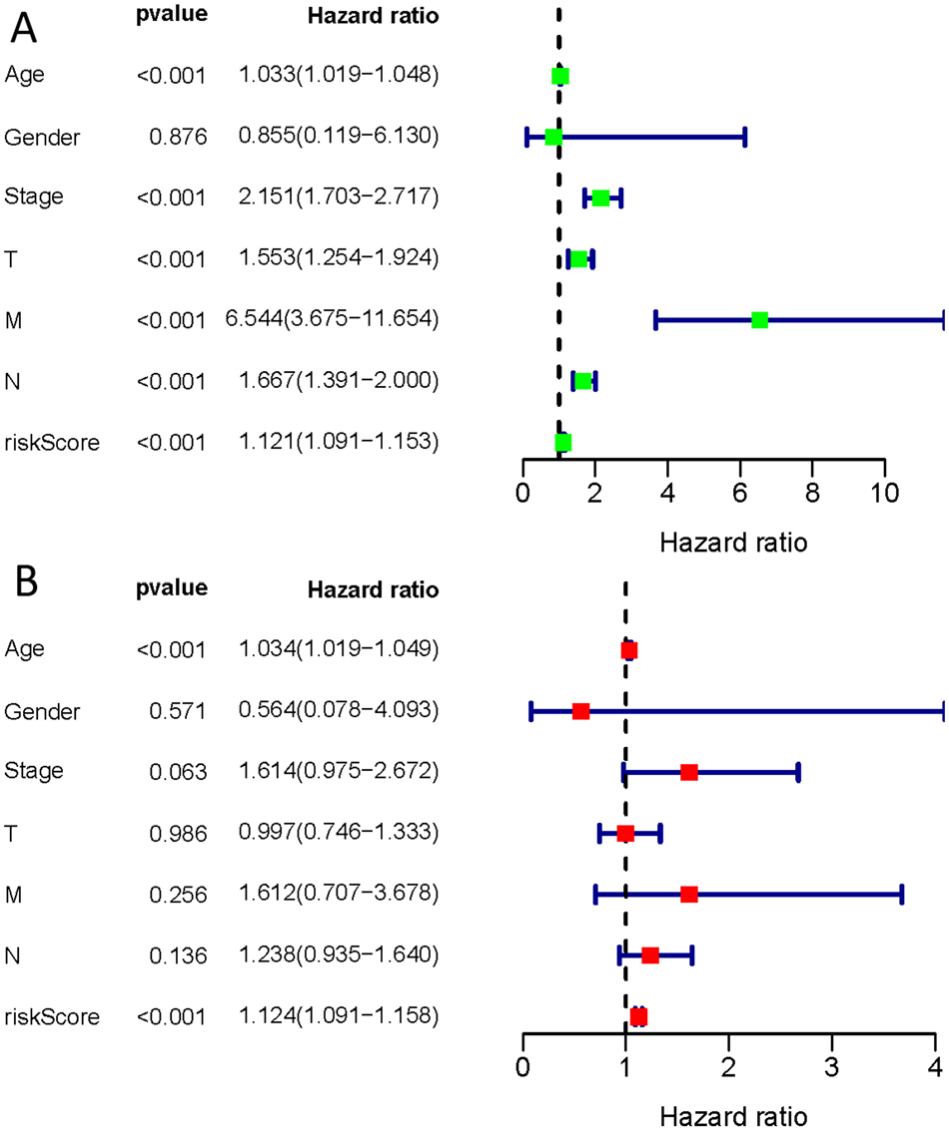
Univariate and multivariate cox proportional hazards model (Hazard Ratio, HR) to identify independent prognostic factors. **A** Univariate cox regression analysis identify of individual factors related to patient survival; **B** Multivariate cox regression analysis identify independent prognostic factors and the result indicated that our signature could as independent prognostic factors for overall survival (HR: 1.124; 95% CI: 1.091–1.158). Green means the value of hazard ratio in univariate and red means the value of hazard ratio in multivariate analysis. (T: Tumor, N: Lymph Node, and M: Metastasis).

**Fig. 4:**
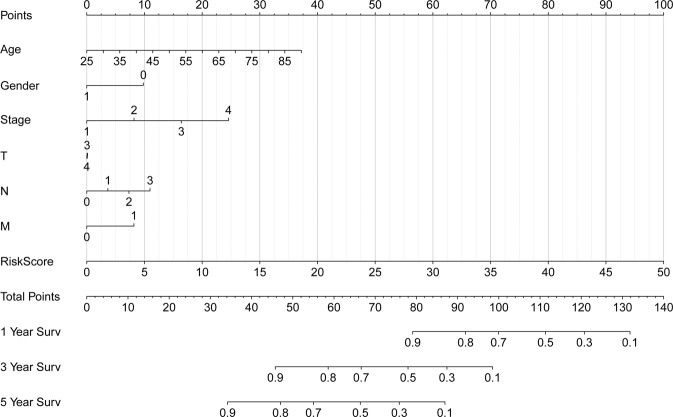
Clinical pathology and prognostic models were used to construct a nomogram through the R language “survival” and “rms” package. The combination enhanced the predictive value of overall survival at 1, 3, and 5 years and, therefore, could be used to inform clinical management.(T: Tumor; N: Lymph Node; and M: Metastasis).

The effect of the expression of 15 ferroptosis genes on patient survival was determined through Kaplan–Meier curve. It was found that the expression of *ALOX15, ANO6, BRD4, CISD1, FLT3, IFNG, NOS2, PROM2*, and *TP63* significantly affected patient’s OS (Table S1). In addition, as shown in Fig. 2B, the Kaplan–Meier curves of the two cohorts established the predictive value of the molecular signature. The high-risk group exhibited a poorer survival outcome when compared to the low-risk group (*p* = 1.645e−13). We also established patient’s risk survival status plot, and as the patient’s risk score increased, the mortality rate also increased (Fig. 2C). Using our molecular signature, we predicted the AUC values of the patients’ 1, 3, 5-year survival rate as 0.74, 0.71, and 0.75, respectively (Fig. 2D). The ROC curves were also used to determine if the expression patterns of survival-related ferroptosis could be used as early markers for the prediction of the occurrence of BC. We found an AUC of 0.719, implying that the sensitivity and specificity of this prognostic model is moderate (Fig. 2E). We then identified risk factors to aid in the establishment of a 15-ferroptosis based prognostic model. The risk score and age were confirmed as independent prognostic factors for OS (Fig. 3). A combination of clinical pathology and prognostic models were used to construct a nomogram (C-index: 0.764). The combination enhanced the predictive value of OS at 1, 3, and 5 years and, therefore, could be used to inform clinical management (Fig. 4). Furthermore, we used GEO to verify the accuracy of our prognostic signature with an AUC of 0.754 (Fig. S1).

### Gene set enrichment analyses

The samples were allocated into high‐ and low‐risk groups to distinguish between their potential functions and elucidate the significant survival differences using GSEA. Most of the metabolic‐related pathways such as citrate cycle tca cycle, glycolysis gluconeogenesis, pentose phosphte pathway, n glycan biosynthesis, propanoate metabolism, o glycan biosynthesis, steroid biosynthesis, starch and sucrose metabolism, biosynthesis of unsaturated fatty acids (Fig. S2 and Table 1).

**Table 1:** Gene sets enriched in phenotype high based on TCGA (Top 10).

Gene set name	SIZE	NES	NOM p-val	FDR q-val
KEGG_CITRATE_CYCLE_TCA_CYCLE	31	2.37	0.000	0.000
KEGG_GLYCOLYSIS_GLUCONEOGENESIS	62	2.11	0.000	0.028
KEGG_PENTOSE_PHOSPHATE_PATHWAY	27	2.07	0.002	0.033
KEGG_N_GLYCAN_BIOSYNTHESIS	46	1.95	0.012	0.069
KEGG_VIBRIO_CHOLERAE_INFECTION	54	1.92	0.000	0.074
KEGG_PROPANOATE_METABOLISM	33	1.90	0.006	0.071
KEGG_O_GLYCAN_BIOSYNTHESIS	30	1.90	0.004	0.064
KEGG_STEROID_BIOSYNTHESIS	17	1.88	0.012	0.063
KEGG_STARCH_AND_SUCROSE_METABOLISM	52	1.82	0.002	0.090
KEGG_BIOSYNTHESIS_OF_UNSATURATED_FATTY_ACIDS	22	1.75	0.026	0.135

*NES* normalized enrichment score, *NOM* nominal, *FDR* false discovery rate.

### Immunity and gene expression

**Fig. 5:**
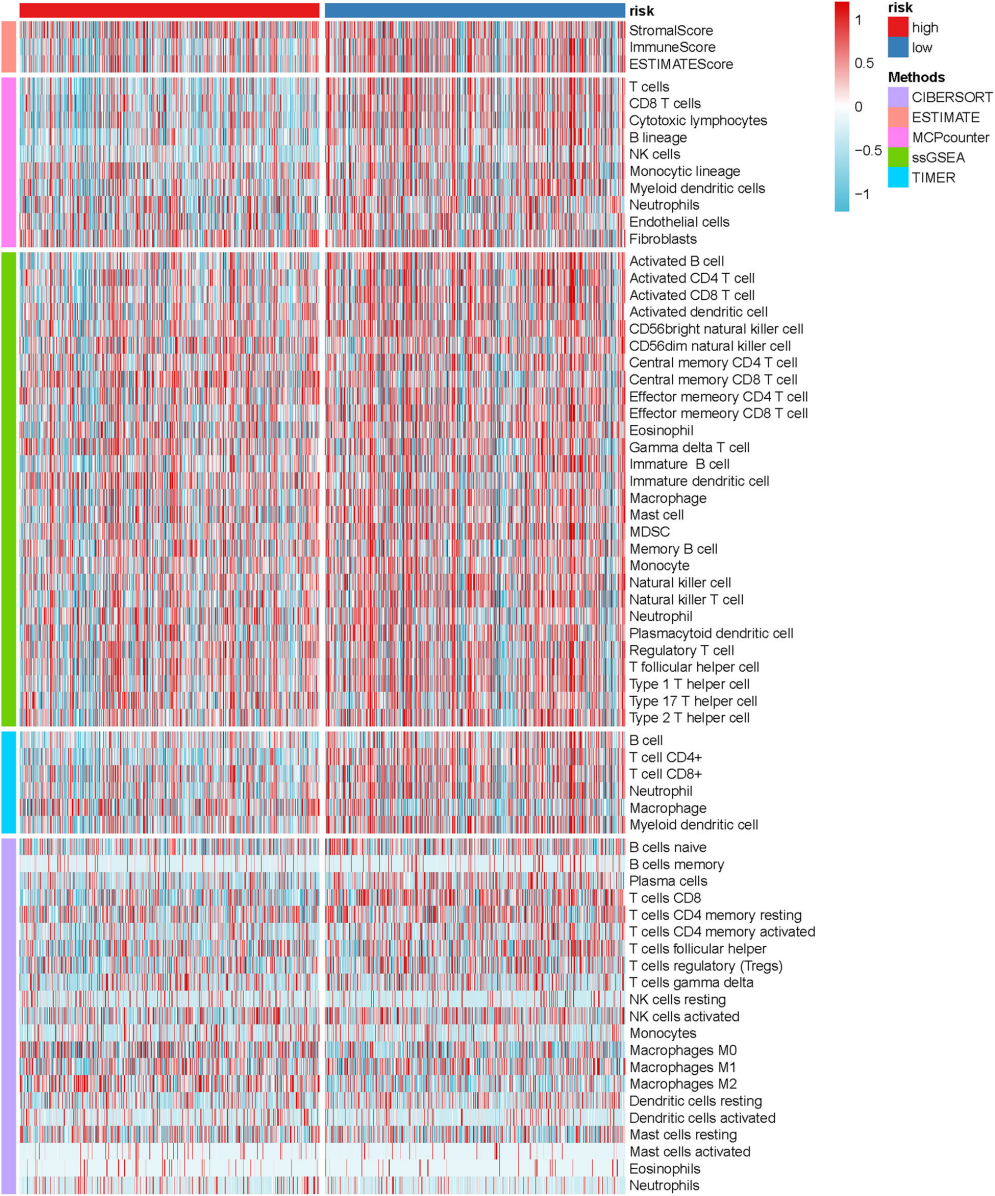
The heatmap of immune responses among high risk and low risk groups based on ferroptosis-related gene signatures. The CIBERSORT, ESTIMATE, MCPcounter, ssGSEA, and TIMER algorithms to assess the cellular components or cell types of immune responses in heterogeneous samples. Red represents high expression and blue represents low expression.

**Fig. 6:**
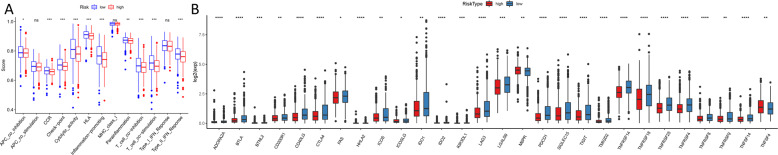
The correlation between immune cell subpopulations and related functions based on ssGSEA in high (red) and low (blue) risk groups, and the differences in the expression levels of immune checkpoints between the high (red) and low (blue) risk groups. **A** Immune function; **B** Immune checkpoints.

The heatmap of immune responses based on five algorithms is shown in (Fig. 5). The correlation between immune cell subpopulations and related functions were evaluated using ssGSEA. T cell functions, including CCR score, immune checkpoint, cytolytic activity, HLA, inflammation promotion, para inflammation, T cell co-stimulation, T cell co-inhibition, and type II INF response were significantly different between the low-risk and high-risk group of the TCGA cohort (Fig. 6A). Studies have characterized the importance of checkpoint inhibitor-based immunotherapies^13^. Therefore, we determined the differences in the expression levels of immune checkpoints between the two groups and found that the expression levels of PDCD-1 (PD-1), CTLA4, LAG3, TNFSF4/14, TNFRSF4/8/9/14/18/25, IDO1/2 et al were significantly different (Fig. 6B).

### miRNA and lncRNA analysis

**Fig. 7:**
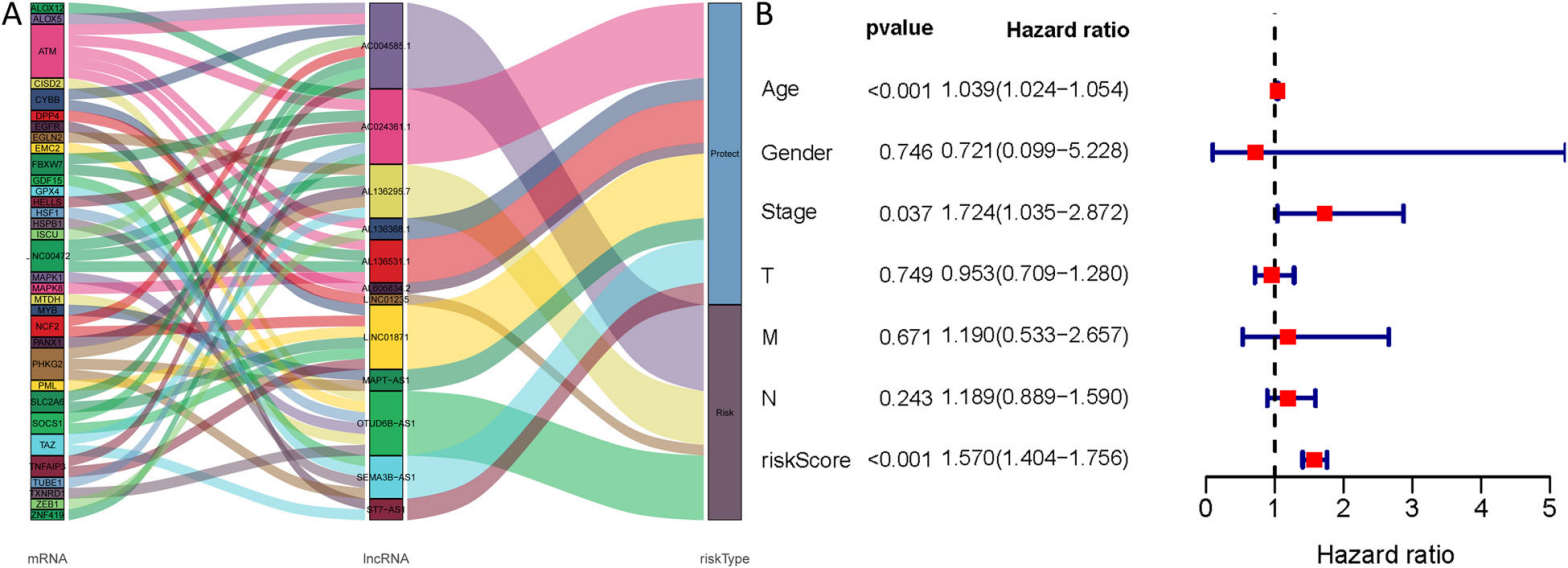
The analysis between the ferroptosis-related genes and lncRNAs based on BC-TCGA. **A** Sankey plots shown the relationship between ferroptosis-related genes and ferroptosis-related lncRNAs; **B** The multivariate COX analysis results based on ferroptosis-related lncRNAs and the result indicated that the lncRNAs signature could as independent prognostic factors for overall survival (HR: 1.570; 95% CI: 1.404–1.756). Red means the value of hazard ratio in multivariate analysis. (T: Tumor, N: Lymph Node, and M: Metastasis).

**Fig. 8:**
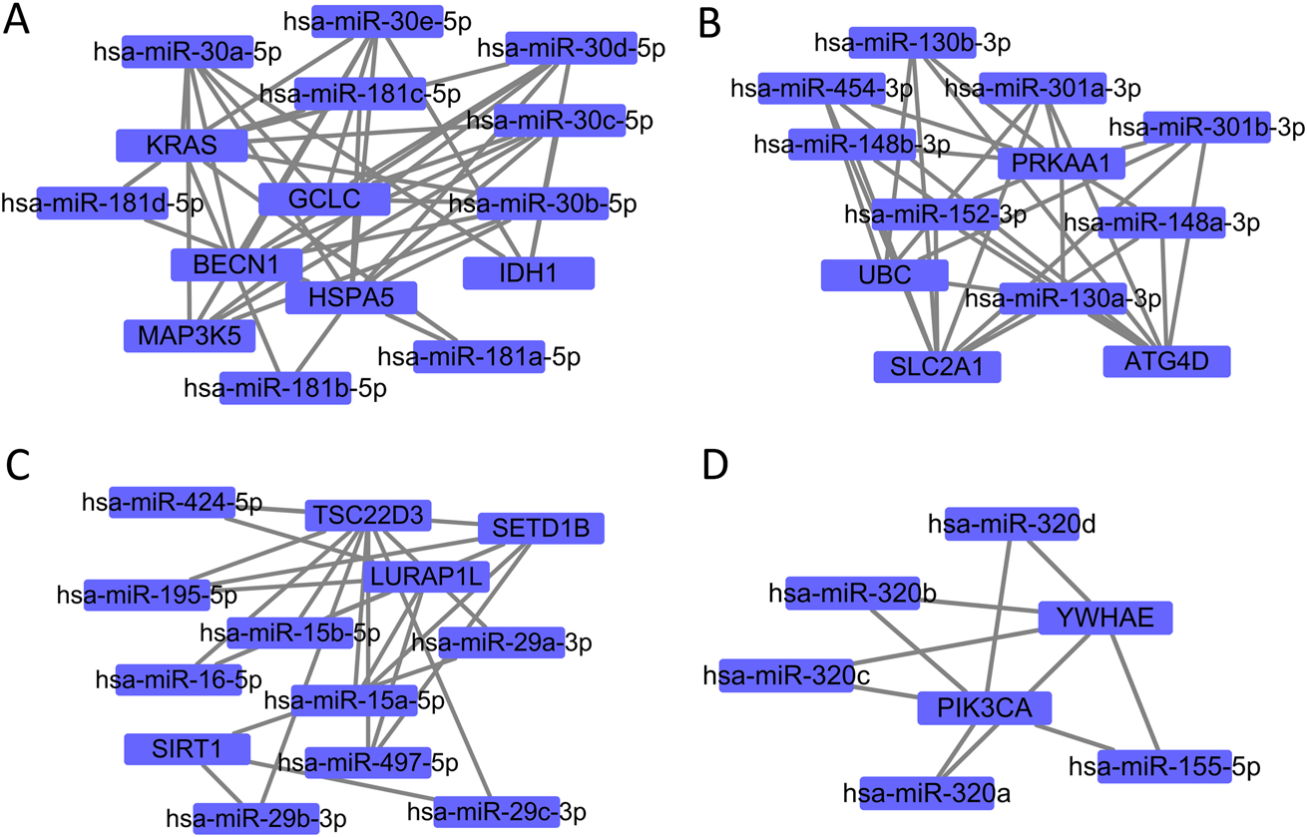
Four densely connected regions in the networks identifying the four hub miRNAs based on the plug-in Molecular Complex Detection (MCODE) of Cytoscape. **A** hsa-miR-181d-5p; **B** hsa-miR-152-3p; **C** hsa-miR-497-5p; **D** hsa-miR-155-5p.

We identified 1185 ferroptosis-related lncRNAs. The relationship between ferroptosis-related genes and lncRNAs is shown in Fig. 7A. Twelve differentially expressed lncRNAs (*AL136531.1, MAPT-AS1, AL606834.2, ST7-AS1, AC024361.1, SEMA3B-AS1, AL136368.1, AC004585.1, LINC01871, AL136295.7, LINC01235*, and *OTUD6B-AS1*) were selected as independent prognostic factors (Table S2). The multivariate COX analysis results demonstrated that the risk score based on ferroptosis-related lncRNAs and age were confirmed as independent prognostic factors for OS (Fig. 7B). The Kaplan–Meier curves, risk survival status plot, and AUC value are presented in Fig. S3. Targetscan^14^ and miranda^15^ database through the ENCORI platform were used to predict the potential binding miRNA of mRNA and only satisfying both two databases been considered potential miRNA, ultimately, 219 ferroptosis-related miRNAs were identified. In addition, Cytoscape was used to establish mRNAs and miRNA networks. The plug-in Molecular Complex Detection (MCODE) of Cytoscape was used to detect densely connected regions in the networks. Four hub miRNAs (hsa-miR-181d-5p, hsa-miR-152-3p, hsa-miR-497-5p, and hsa-miR-155-5p) were identified in the network (Fig. 8).

## Discussion

Ferroptosis can eliminate the adaptive characteristics of malignant cells, remove cells that cannot obtain key nutritional factors, are infected or damaged by environmental changes. It plays a key role in inhibiting tumorigenesis and is a potential strategy for tumor treatment. In this study, we identified a novel and useful ferroptosis-related prognostic gene signature based on the TCGA dataset. The signature was validated in the GSE7390 dataset of BC patients. Meanwhile, we also evaluated the role of ferroptosis-related miRNAs and constructed a ferroptosis-related lncRNAs signature. Potential immune responses involving the tumor microenvironment, tumor-infiltrating immune cells, immune function, and immune checkpoints were also evaluated. Therefore, this study informs on the potential biomarkers and therapeutic targets of the ferroptosis signaling pathways.

Analysis of the 259 ferroptosis-related genes and their potential functions revealed that KEGG were mainly enriched in the HIF-1 signaling pathway, NOD-like receptor signaling pathway, central carbon metabolism in cancer, and PPAR signaling pathway. It has been reported that the efficacy of FG-4592 (an inhibitor of prolyl hydroxylase of HIF) pretreatment is attained by suppressing ferroptosis in the advanced stages of kidney injury through Akt/GSK-3β-mediated Nrf2 activation^16^. In addition, Yu et al.^17^ reported the correlation between central carbon catabolic pathways and ferroptosis in HNSCC cisplatin resistance. PPARα activity is essential for the promotion of ferroptosis in cancer by MDM2 and MDMX (tumor suppressor p53 negative regulators)^18^. In this study, 15 ferroptosis genes (*ALOX15, ALOX15B, ANO6, BRD4, CISD1, DRD5, FLT3, G6PD, IFNG, NGB, NOS2, PROM2, SLC1A4, SLC38A1*, and *TP63*) were selected as independent prognostic factors for BC. By up-regulating *GPX4* and down-regulating arachidonic 15-lipoxygenase (*ALOX15*), the silenced GSK-3 protein blocked erastin-induced ferroptosis and secreted few ROS molecules in BC. The role for *ALOX15B* (*ALOX15* type B), and to a lesser extent *ALOX15*, in cholesterol homeostasis in human macrophages have been documented^19^. *ANO6* (*TMEM16F*) serves as a Ca^2+^ activated chloride and scrambles membrane phospholipids to disclose phosphatidylserine at the cell surface. Activation of *ANO6* is essential during ferroptosis mediated cancer cell death^20^. A high expression of BRD4 in cancer tissues has been associated with poor prognosis. Ferroptosis was found to be induced by *BRD4* knockdown^21^. *CISD1* (mitoNEET), an iron-containing outer mitochondrial membrane protein, negatively regulates ferroptosis cancer cell death, and promotes the proliferation of cancer cells, adjuvant tumor growth and metastasis^22,23^. Dopamine receptor D1-like receptors (*DRD5*) regulates tumor behavior^24^. FLT3 inhibitors, as potent protectors, were found to inhibit glutamate toxicity and ROS generation^25^. *G6PD* is associated with mitochondrial dysfunction^26^ and human neuroglobin. It binds proteins of the cellular iron metabolism under ferroptosis stress^27^. Prominin2 (*PROM2*) can suppress ferroptosis sensitivity^28^. *SLC38A1* which is an important regulator of lipid peroxidation^29^ and *TP63* gene polymorphism enhances the risk of BC development^30^.

Five immune algorithms were then used to assess the relative immune cell infiltrations of each sample, and therefore, establish the efficacy of gene signatures for predicting the OS of BC patients. Our prognostic signature was found to be a reliable tool for predicting the prognosis of BC patients. The induction of ferroptosis combined with immune checkpoint inhibitors (ICIs) showed a synergistically enhanced antitumor activity, even in ICI-resistant tumors^31^. A limited number of studies have evaluated the relationship between ICI and ferroptosis. This study provides important avenues for future research, especially *PD-1* and *CTLA4* targets.

microRNA (miRNA) and long non-coding RNA (lncRNA) are key mediators for regulating ferroptosis. Twelve ferroptosis-related lncRNAs were used to build the lncRNA signature. Four hub miRNAs that could be associated with ferroptosis were identified. LINC00618 promotes ferroptosis by elevating lipid ROS and iron levels, and by suppressing the expression levels of solute carrier family 7 member 11 (*SLC7A11*) in human leukemia^32^. lncRNA ZFAS1 knockdown attenuates ferroptosis by sponging miR-150-5p to downregulate *SLC38A1* expression in pulmonary fibrosis^29^. Since Nrf2 inhibits iron absorption, limits ROS production and up-regulates the expression of *SLC7A11*, miRNA can affect ferroptosis by regulating the Nrf2 expression^33–35^. miRNA is also involved in the regulation of iron transport, storage, utilization and absorption. It has been documented that miR-20a regulates the expression of iron exporter ferroportin (FPN) and decreases iron export, leading to intracellular iron retention, which in turn favors cell proliferation in lung cancer^36^.

Ferroptosis, as a form of cell death provides a new therapeutic strategy for tumor treatment. However, various issues such as the interconnection between ferroptosis and other cell deaths, and host immunogenicity have not been established. In this study, we integrated ferroptosis biomarkers to assess treatment outcomes and to reveal precise therapeutic targets for BC. However, more studies using independent cohorts and predictive ferroptosis functional experiments should be performed to validate our signature. The limitations of our study are that; our outcomes were not clinically validated and the insufficient data from the small sample size limits the statistical power of our findings.

In summary, we identified novel ferroptosis-related biomarkers markers for BC prognosis. This study provides novel insights for the development of reliable and accurate cancer treatment tools.

## Materials and methods

### Data collection

RNA-seq (113 normal and 1109 tumor) data and miRNAs-seq (104 normal and 1103 tumor) data for BC patients was obtained from TCGA. The RNA-seq data of the GSE7390 (198 tumor tissues) dataset obtained from the GEO database was validated and its clinical characterizes presented in Table 2. Ferroptosis-related genes were obtained from FerrDb, a web-based resource. FerrDb provides a comprehensive and up-to-date list of ferroptosis markers, regulatory molecules and ferroptosis-disease associations^37^. We obtained 259 (Driver: 108; suppressor: 69; marker: 111) ferroptosis-related genes. Pearson correlation analysis was used to determine the correlations among ferroptosis-related genes and lncRNAs. A ferroptosis-related lncRNA association was considered positive with a correlation coefficient |*R*^*2*^| > 0.3 and *p* < 0.001.

**Table 2:** TCGA and GEO breast cancer patient characteristics.

		TCGA	GEO
Clinical characteristics		Total (1097)^a^	Total (198)
Age at diagnosis (y)		47 (26–90)	48 (24–60)
Gender	Female	1085	198
	Male	12	0
Stage	I	184	NA
	II	622	NA
	III	247	NA
	IV	20	NA
	NA	24	NA
Grade	1	NA	30
	2	NA	83
	3	NA	83
	NA	NA	2
T-classification	T1	281	25
	T2	635	41
	T3	137	13
	T4	41	NA
	NA	3	119
M-classification	M0	912	NA
	M1	22	NA
	MX	163	NA
N-classification	N0	516	198
	N1	365	NA
	N2	119	NA
	N3	77	NA
	NX	20	NA
Status	Alive	948	142
	Death	149	56

Data express as Mean (min–max).

^a^The remaining value after removing the sample with missing data from the total number of people.

The collected clinicopathological data included gender, age, stage, TMN classification, survival status and the number of survival days. FDR < 0.05 and |log_2_FC| ≥ 1 were determined as differentially expressed ferroptosis-related genes. Moreover, we evaluated the functions of the identified ferroptosis-related genes using Gene ontology (GO) function analysis (biological processes (BP), molecular functions (MF), and cellular components (CC)) and Kyoto Encyclopedia of Genes and Genomes (KEGG) through the R language ggplot2 package.

### Development of the ferroptosis-related prognostic gene signature

Lasso‐penalized Cox regression and Univariate Cox regression analyses were used to build the ferroptosis-related prognostic gene signature. The regression coefficient β value (coef) of each gene is obtained in the Cox model, and the positive sign indicates that the risk (mortality) is higher, so the prognosis is worse. The signature was defined as risk score = (CoefficientmRNA1 × expression of mRNA1) + (CoefficientmRNA2 × expression of mRNA2) + ⋯ + (CoefficientmRNAn × expression mRNAn). miRNA-based signature and lncRNA-based signature were also used the same formula to calculate. The associated clinical data for BC patients were also downloaded and evaluated. Based on the median, we denoted the data as either low-risk (<median number) or high-risk (≥median number) group. The Kaplan–Meier survival analysis was used to determine the survival rate.

### Building a predictive nomogram

We developed a nomogram for predicting the occurrence of cancer events, such as recurrence or death. The time-dependent receiver operating characteristic (ROC) curve was used to evaluate the predictive accuracy of the developed prognostic signatures for BC patients. Univariate/multivariate Cox regression analyses were used to evaluate the connection between ferroptosis-related genes and clinicopathological manifestations. To understand the mechanisms underlying the gene signatures in the KEGG pathways, we used Gene set enrichment analyses (GSEA) to search for rich terms in C2 in the TCGA-BC cohort. *P* < 0.05 and false discovery rate (FDR) *q* < 0.05 were considered to be statistically significant.

### Immune analysis and gene expression”

We compared CIBERSORT^38,39^, ESTIMATE^40^, MCPcounter^41^, single-sample gene set enrichment analysis (ssGSEA)^42^, and TIMER^43^ algorithms to assess the CC or cell types of immune responses in heterogeneous samples among high-risk and low-risk groups based on ferroptosis-related gene signatures. ssGSEA was then used to quantify tumor-infiltrating immune cell subgroups during immune responses and immune functions between the two groups. Potential immune checkpoints were extracted from previous literature.

### Statistical analysis

Statistical analyses were performed using R version 3.5.3 and Bioconductor. The unpaired Student’s *t* test and the Wilcoxon test were used to evaluate the normal distribution variables and the non-normal distribution variables, respectively. To identify differential genes in DEGs assessments, the Benjamini–Hochberg method was used to convert the *p* value to FDR. ssGSEA-normalized BC DEGs data were compared to a genome using “GSVA” (R-package). For each statistical analysis, a *p* ≤ 0.05 was considered to be statistically significant.

## Supplementary information

Table S1

Table S2

UPPLEMENTAL MATERIAL

UPPLEMENTAL MATERIAL

UPPLEMENTAL MATERIAL

UPPLEMENTAL MATERIAL

## Data Availability

Data sharing is not applicable to this article as no datasets were generated or analyzed during the current study.
