# Supplementary material for: The role of ferroptosis in breast cancer patients: a comprehensive analysis
Source: Cell Death Discov. 2021 May 4;7:93. doi: 10.1038/s41420-021-00473-5 (PMC8097021; doi:10.1038/s41420-021-00473-5)
Supplement: Supplementary file 1 — Table S1 [file 41420_2021_473_MOESM1_ESM.docx]

| Univariate COX analysis |  |  |  |  | Multivariate Cox analysis |  |  |  |  |  |  |  |
| --- | --- | --- | --- | --- | --- | --- | --- | --- | --- | --- | --- | --- |
| id | HR | HR.95L | HR.95H | pvalue | id | coef | HR | HR.95L | HR.95H | pvalue | P value （overall survival） | Sig |
| ACSF2 | 0.804096409 | 0.680543058 | 0.950081008 | 0.010419824 | ALOX15 | 0.176756612 | 1.193340613 | 0.978580267 | 1.455232511 | 0.08079839 | 5.92E-03 | ** |
| ALOX15 | 1.283996918 | 1.073832849 | 1.535293027 | 0.006124032 | ALOX15B | 0.108545493 | 0.89713808 | 0.81518702 | 0.987327711 | 0.026357521 | 8.35E-02 |  |
| ALOX15B | 0.903858413 | 0.819506608 | 0.996892548 | 0.043152851 | ANO6 | 0.53476043 | 1.707039237 | 1.224806343 | 2.37913771 | 0.001593065 | 2.05E-02 | * |
| ANO6 | 1.421340923 | 1.070602335 | 1.886984509 | 0.015023568 | BRD4 | 0.704552872 | 0.494329554 | 0.332781314 | 0.734301167 | 0.000483722 | 1.02E-02 | * |
| BNIP3 | 1.288591542 | 1.035196573 | 1.604012422 | 0.023231768 | CISD1 | 0.473056335 | 1.604891793 | 1.184299164 | 2.174853909 | 0.002281837 | 7.95E-03 | ** |
| BRD4 | 0.518341614 | 0.342933704 | 0.7834693 | 0.001822392 | DRD5 | 3.530279662 | 34.13351211 | 2.834690297 | 411.013736 | 0.005425026 | 1.76E-01 |  |
| CHMP6 | 0.718705206 | 0.538740103 | 0.958787309 | 0.024693711 | FLT3 | 0.160325829 | 0.851866181 | 0.697652199 | 1.040168714 | 0.11561337 | 1.44E-02 | * |
| CISD1 | 1.555159696 | 1.173618686 | 2.060738901 | 0.002107362 | G6PD | 0.188702968 | 1.207682179 | 0.970524965 | 1.502791065 | 0.09069475 | 1.77E-01 |  |
| CS | 1.667673811 | 1.159690921 | 2.398169969 | 0.005792738 | IFNG | 0.540568755 | 0.582416906 | 0.371283828 | 0.913612247 | 0.018608368 | 8.60E-04 | *** |
| DRD5 | 10.73114063 | 1.053084189 | 109.3524908 | 0.045109335 | NGB | 0.333249119 | 1.3954949 | 0.953972143 | 2.041365704 | 0.085949823 | 9.83E-01 |  |
| EMC2 | 1.655760577 | 1.248679006 | 2.195554722 | 0.00046081 | NOS2 | 0.671608278 | 1.957382806 | 1.232808928 | 3.107819355 | 0.004409654 | 1.80E-02 | * |
| FLT3 | 0.744105448 | 0.614789691 | 0.900621669 | 0.002408602 | PROM2 | 0.260201985 | 1.297192074 | 1.040891659 | 1.616601749 | 0.020513986 | 4.08E-02 | * |
| G6PD | 1.261946735 | 1.035789997 | 1.537483048 | 0.020946539 | SLC1A4 | 0.289126417 | 0.748917523 | 0.616783842 | 0.90935822 | 0.003507526 | 1.28E-01 |  |
| GCLC | 1.320282362 | 1.008764783 | 1.727999972 | 0.043019687 | SLC38A1 | 0.15915978 | 1.172525278 | 0.963824659 | 1.426416635 | 0.111492032 | 7.26E-02 |  |
| IFNG | 0.609113218 | 0.40574455 | 0.914415023 | 0.016775768 | TP63 | 0.242098346 | 0.784978974 | 0.652260763 | 0.944701911 | 0.010408815 | 1.93E-02 | * |
| IL33 | 0.84701286 | 0.738142808 | 0.971940358 | 0.018009578 |  |  |  |  |  |  |  |  |
| JUN | 0.823560021 | 0.701501064 | 0.966856849 | 0.017702869 |  |  |  |  |  |  |  |  |
| MAP1LC3A | 0.815508694 | 0.674939912 | 0.985353538 | 0.034616064 |  |  |  |  |  |  |  |  |
| MIR30B | 2.301350425 | 1.156371063 | 4.58002967 | 0.017609487 |  |  |  |  |  |  |  |  |
| MTDH | 1.423180845 | 1.140201205 | 1.77639149 | 0.001808827 |  |  |  |  |  |  |  |  |
| NGB | 1.651077292 | 1.211004312 | 2.251070618 | 0.001521841 |  |  |  |  |  |  |  |  |
| NOS2 | 2.07274884 | 1.317683134 | 3.260486259 | 0.001612857 |  |  |  |  |  |  |  |  |
| PANX1 | 1.507215468 | 1.102167182 | 2.061119677 | 0.010195227 |  |  |  |  |  |  |  |  |
| PIK3CA | 1.457347279 | 1.085302762 | 1.956929593 | 0.012270241 |  |  |  |  |  |  |  |  |
| PROM2 | 1.311680371 | 1.058386455 | 1.625592794 | 0.013200455 |  |  |  |  |  |  |  |  |
| SLC1A4 | 0.826605715 | 0.69672876 | 0.980692987 | 0.028998259 |  |  |  |  |  |  |  |  |
| SLC38A1 | 1.177351697 | 1.000135814 | 1.385968784 | 0.049809582 |  |  |  |  |  |  |  |  |
| SLC7A5 | 1.115828101 | 1.001543736 | 1.243153251 | 0.046817911 |  |  |  |  |  |  |  |  |
| SOCS1 | 0.809501542 | 0.667543062 | 0.981648652 | 0.031694815 |  |  |  |  |  |  |  |  |
| TNFAIP3 | 0.834517319 | 0.70126413 | 0.993091084 | 0.041542815 |  |  |  |  |  |  |  |  |
| TP63 | 0.79230109 | 0.666770537 | 0.941464841 | 0.008161257 |  |  |  |  |  |  |  |  |
| TXNRD1 | 1.247960144 | 1.015680243 | 1.533361049 | 0.03502848 |  |  |  |  |  |  |  |  |
| VDAC2 | 1.471470958 | 1.063980243 | 2.035025362 | 0.019551976 |  |  |  |  |  |  |  |  |
