# Supplementary material for: The role of ferroptosis in breast cancer patients: a comprehensive analysis
Source: Cell Death Discov. 2021 May 4;7:93. doi: 10.1038/s41420-021-00473-5 (PMC8097021; doi:10.1038/s41420-021-00473-5)
Supplement: Supplementary file 2 — Table S2 [file 41420_2021_473_MOESM2_ESM.docx]

| Univariate COX analysis |  |  |  |  |  |  |  | Multivariate Cox analysis |  |  |
| --- | --- | --- | --- | --- | --- | --- | --- | --- | --- | --- |
| gene | KM | B | SE | HR | HR.95L | HR.95H | pvalue | id | coef | HR |
| AC026471.4 | 0.036201586 | -0.067701703 | 0.031746673 | 0.934539202 | 0.878162134 | 0.994535617 | 0.032960791 | AL136531.1 | -0.327569644 | 0.720673099 |
| BAIAP2-DT | 0.003085026 | -0.049760336 | 0.024872918 | 0.951457428 | 0.906186306 | 0.998990198 | 0.045437351 | MAPT-AS1 | -0.326995319 | 0.721087119 |
| USP30-AS1 | 0.007527661 | -0.22245823 | 0.085664456 | 0.800548445 | 0.676814884 | 0.946902658 | 0.009408144 | AL606834.2 | -0.306950238 | 0.735687209 |
| AL136531.1 | 0.01655112 | -0.398357792 | 0.197426781 | 0.671421756 | 0.455979616 | 0.988656418 | 0.043617355 | ST7-AS1 | -0.396432786 | 0.672715491 |
| AC103691.1 | 0.025371028 | -0.200359593 | 0.096341973 | 0.818436396 | 0.677607958 | 0.988533452 | 0.037555734 | AC024361.1 | -0.326951038 | 0.72111905 |
| MAPT-AS1 | 0.000966006 | -0.328976764 | 0.098533869 | 0.719659738 | 0.593273603 | 0.872970139 | 0.000841661 | SEMA3B-AS1 | -0.076561511 | 0.926295935 |
| AL606834.2 | 0.003770787 | -0.380268735 | 0.135124683 | 0.683677656 | 0.524605751 | 0.890983632 | 0.004889784 | AL136368.1 | -0.345195776 | 0.708081715 |
| LINC00852 | 0.024668031 | -0.479391153 | 0.243741279 | 0.619160251 | 0.383999018 | 0.998334364 | 0.049205892 | AC004585.1 | 0.195761678 | 1.216237014 |
| ST7-AS1 | 0.001761865 | -0.638942035 | 0.191970257 | 0.527850576 | 0.362331107 | 0.768982362 | 0.000873656 | LINC01871 | -0.347333152 | 0.706569894 |
| AC136475.2 | 0.010121947 | -0.165285543 | 0.078808912 | 0.847651629 | 0.726331924 | 0.989235444 | 0.035967464 | AL136295.7 | 0.201821942 | 1.223630111 |
| AL645924.1 | 0.02746684 | -0.227586911 | 0.114345222 | 0.796453198 | 0.636545469 | 0.996531634 | 0.046552491 | LINC01235 | 0.009120989 | 1.009162712 |
| AC005840.4 | 0.038197088 | -0.335349846 | 0.161132755 | 0.715087872 | 0.521438342 | 0.980654131 | 0.037415401 | OTUD6B-AS1 | 0.052406768 | 1.053804309 |
| AP001160.3 | 0.019373322 | -0.200258245 | 0.081546194 | 0.818519347 | 0.697616435 | 0.960375771 | 0.014058526 |  |  |  |
| LINC01786 | 0.047828453 | -0.257651364 | 0.127371393 | 0.772864634 | 0.602122294 | 0.992023959 | 0.04309012 |  |  |  |
| PRR34-AS1 | 0.049237352 | -0.088891443 | 0.040640912 | 0.914944892 | 0.844892257 | 0.990805808 | 0.028724982 |  |  |  |
| STAG3L5P-PVRIG2P-PILRB | 0.005143505 | -0.381031055 | 0.157356943 | 0.683156674 | 0.501854523 | 0.929956828 | 0.015458974 |  |  |  |
| AC024361.1 | 0.00222471 | -0.449103525 | 0.206786781 | 0.638200025 | 0.425539217 | 0.957136867 | 0.029869294 |  |  |  |
| AC087741.1 | 0.003197605 | -0.2209138 | 0.09531079 | 0.801785791 | 0.665165421 | 0.966467039 | 0.020458795 |  |  |  |
| AP005131.2 | 0.002272409 | -0.3741083 | 0.172765395 | 0.687902408 | 0.490307639 | 0.965128189 | 0.03035613 |  |  |  |
| SEMA3B-AS1 | 0.023814537 | -0.079385112 | 0.030144757 | 0.923684134 | 0.870691331 | 0.979902234 | 0.008451897 |  |  |  |
| NIFK-AS1 | 0.000353471 | -0.421596751 | 0.145666277 | 0.655998517 | 0.493073313 | 0.872758762 | 0.003800477 |  |  |  |
| AL136368.1 | 0.003310209 | -0.620027549 | 0.231785059 | 0.537929618 | 0.341530628 | 0.847268882 | 0.007472683 |  |  |  |
| AC004585.1 | 0.005012819 | -0.177177281 | 0.089725419 | 0.837631276 | 0.702551992 | 0.998682179 | 0.048306638 |  |  |  |
| AC020907.4 | 0.007126692 | -0.324543149 | 0.146651946 | 0.722857517 | 0.542278441 | 0.963569542 | 0.026896521 |  |  |  |
| DLG5-AS1 | 0.019221085 | -0.186759675 | 0.074557925 | 0.829643096 | 0.716848668 | 0.960185459 | 0.012248821 |  |  |  |
| NDUFV2-AS1 | 0.002590036 | -0.3722459 | 0.187887118 | 0.689184751 | 0.476876427 | 0.996014048 | 0.047566497 |  |  |  |
| LINC01871 | 0.01949782 | -0.217804459 | 0.064494717 | 0.804282697 | 0.708778806 | 0.912655191 | 0.000732571 |  |  |  |
| AL109811.2 | 0.033233849 | -0.157049627 | 0.069474264 | 0.854661644 | 0.745860509 | 0.979333959 | 0.023787512 |  |  |  |
| AL136295.7 | 0.012580212 | -0.196932931 | 0.092386805 | 0.821245711 | 0.685225196 | 0.984266957 | 0.033038659 |  |  |  |
| SH3BP5-AS1 | 0.001457999 | -0.266410997 | 0.102046976 | 0.766124188 | 0.627244166 | 0.935754054 | 0.0090365 |  |  |  |
| AL358472.3 | 0.031511437 | -0.298340289 | 0.108946184 | 0.742048786 | 0.599373171 | 0.918687101 | 0.006173538 |  |  |  |
| AC010201.2 | 0.001083055 | -0.536454266 | 0.239084656 | 0.58481819 | 0.366025754 | 0.934394128 | 0.024846318 |  |  |  |
| AC004067.1 | 0.033349589 | -0.385985056 | 0.184191318 | 0.679780684 | 0.473788904 | 0.97533263 | 0.036120711 |  |  |  |
| AC254562.3 | 0.021802205 | -0.366877937 | 0.183556832 | 0.692894217 | 0.483529614 | 0.992912081 | 0.045639114 |  |  |  |
| LINC01235 | 0.019505663 | 0.011029047 | 0.004631521 | 1.011090091 | 1.001953338 | 1.020310161 | 0.01725159 |  |  |  |
| AC107464.2 | 0.005410923 | -0.174843952 | 0.07164958 | 0.839588027 | 0.72958854 | 0.966172049 | 0.014676497 |  |  |  |
| AC004975.2 | 0.046447322 | -0.433736608 | 0.188296267 | 0.648082933 | 0.448076855 | 0.937364836 | 0.021251874 |  |  |  |
| TNFRSF14-AS1 | 0.000878762 | -0.57603651 | 0.195910124 | 0.562121922 | 0.382887795 | 0.82525758 | 0.003278839 |  |  |  |
| OTUD6B-AS1 | 0.016614479 | 0.075298226 | 0.026462094 | 1.078205652 | 1.023710197 | 1.13560208 | 0.004433998 |  |  |  |
| U73166.1 | 0.013693056 | -0.349047763 | 0.174235854 | 0.705359439 | 0.501303405 | 0.992476679 | 0.045144435 |  |  |  |
| EGOT | 0.006558999 | -0.135512107 | 0.061648595 | 0.87326859 | 0.773877929 | 0.985424189 | 0.027939286 |  |  |  |
| PCED1B-AS1 | 0.00326661 | -0.105773178 | 0.048505095 | 0.899628681 | 0.818042149 | 0.989352155 | 0.029208485 |  |  |  |
| ERICH6-AS1 | 0.024938231 | -0.218521068 | 0.101705023 | 0.803706546 | 0.658454899 | 0.980999935 | 0.03166795 |  |  |  |
| AC234582.1 | 0.000754247 | -0.280770432 | 0.123798086 | 0.755201686 | 0.592496554 | 0.962587178 | 0.023330992 |  |  |  |
