# Supplementary figures and images for: The role of ferroptosis in breast cancer patients: a comprehensive analysis
Source: Cell Death Discov. 2021 May 4;7:93. doi: 10.1038/s41420-021-00473-5 (PMC8097021; doi:10.1038/s41420-021-00473-5)

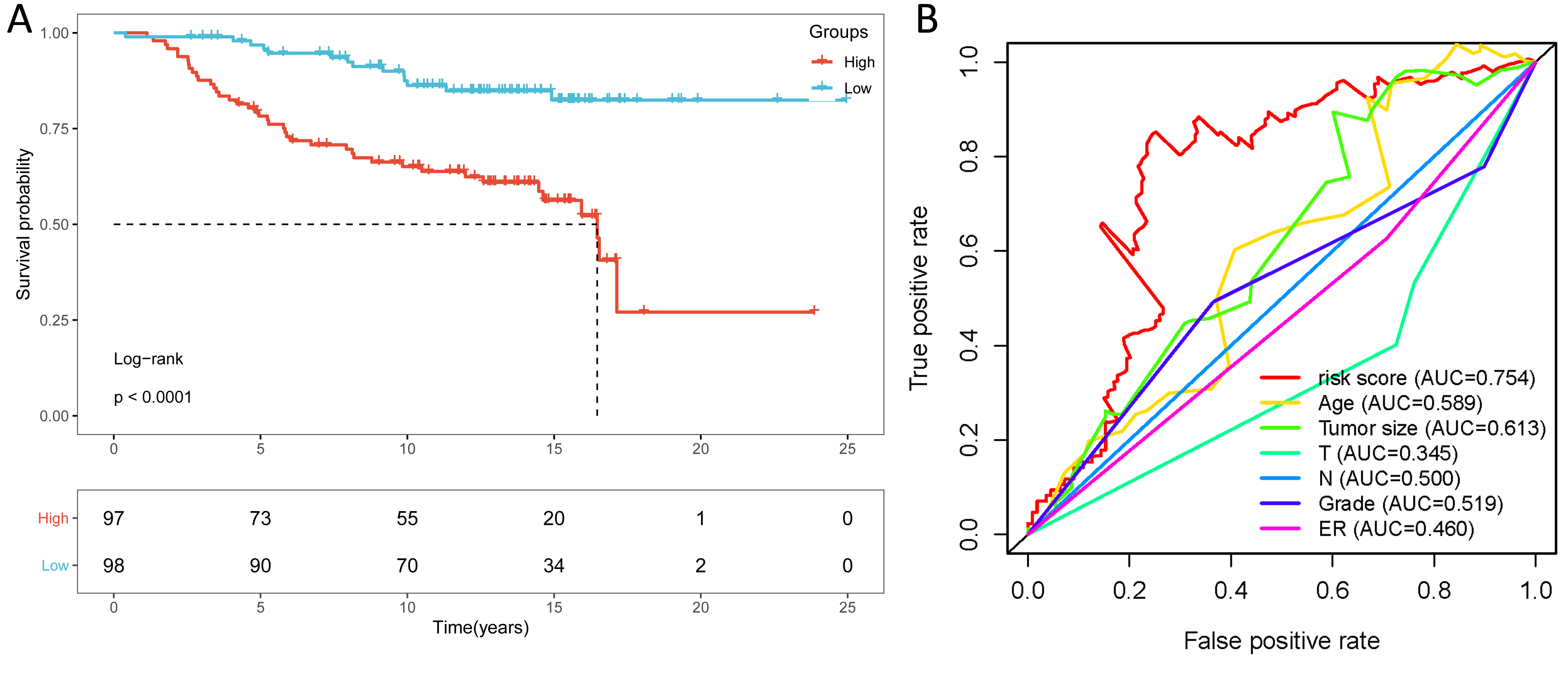

Supplement: Supplementary file 3 — UPPLEMENTAL MATERIAL [file 41420_2021_473_MOESM3_ESM.tif]

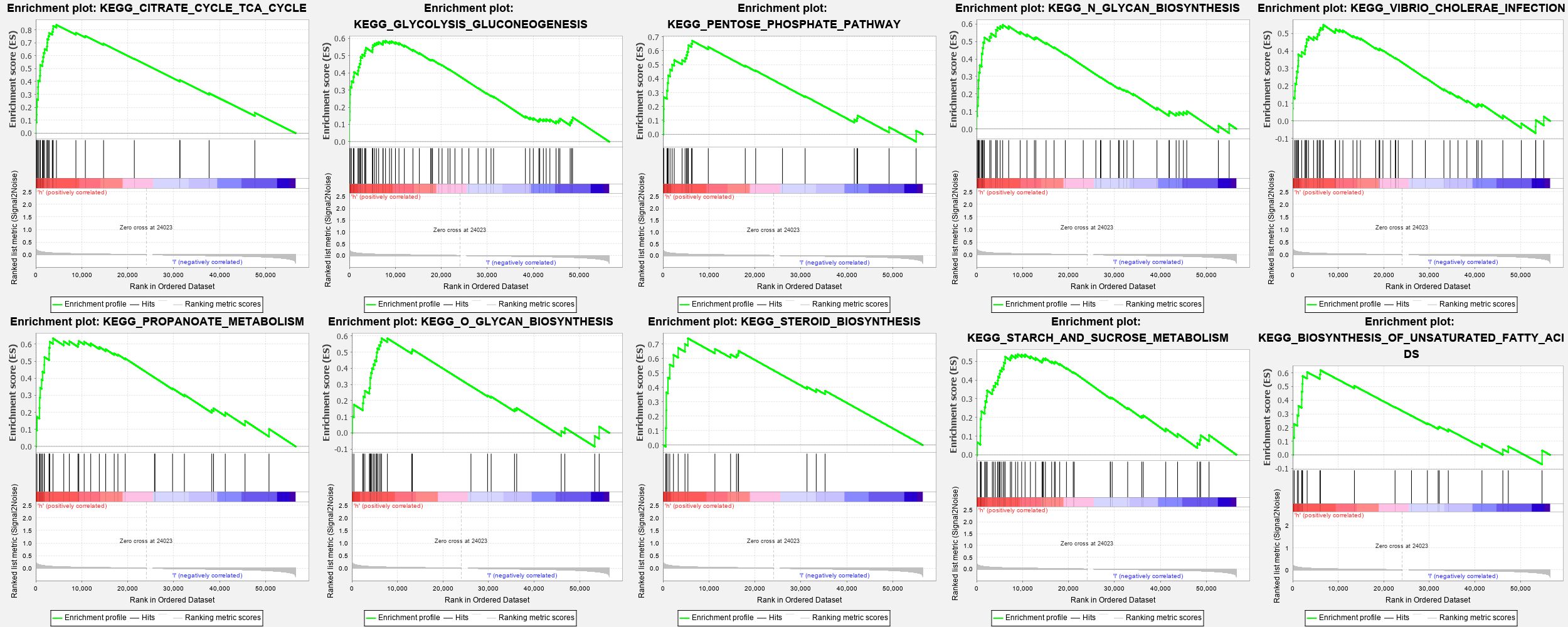

Supplement: Supplementary file 4 — UPPLEMENTAL MATERIAL [file 41420_2021_473_MOESM4_ESM.tif]

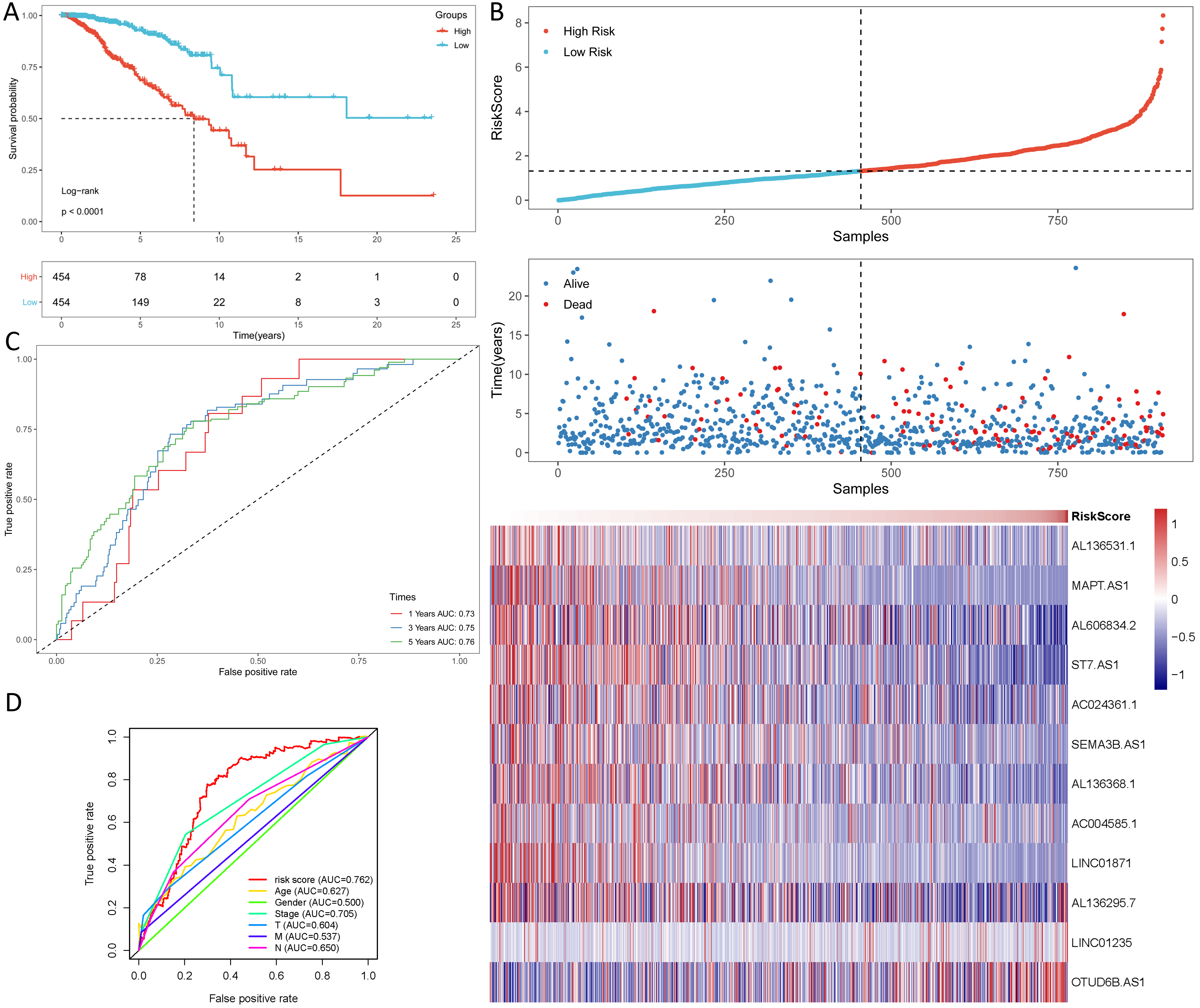

Supplement: Supplementary file 5 — UPPLEMENTAL MATERIAL [file 41420_2021_473_MOESM5_ESM.tif]
