# Supplementary material for: The role of ferroptosis in breast cancer patients: a comprehensive analysis
Source: Cell Death Discov. 2021 May 4;7:93. doi: 10.1038/s41420-021-00473-5 (PMC8097021; doi:10.1038/s41420-021-00473-5)
Supplement: Supplementary file 6 — UPPLEMENTAL MATERIAL [file 41420_2021_473_MOESM6_ESM.docx]

**Figure S1**: Evaluate the accuracy of the ferroptosis-related gene signature based on GEO (Gene Expression Omnibus database). **A**. Kaplan-Meier indicated that high-risk group (red) exhibited a poorer survival outcome when compared to the low-risk group (blue); **B.** the AUC of the signature was 0.754, exhibiting superior performance than the traditional clinicopathological features in predicting the prognosis.

**Figure S2**: Gene set enrichment analyses to distinguish potential functions and elucidate the significant survival differences in phenotype high risk groups based on TCGA.

**Figure S3**: Evaluate the accuracy of the ferroptosis-related lncRNAs signature based on TCGA. **A**. Kaplan-Meier indicated that high-risk group (red) exhibited a poorer survival outcome when compared to the low-risk group (blue); **B.** The top graph represents the classification of patients into high (red) and low (blue) risk groups based on risk scores, the middle graph represents as the patient's risk score increased, the mortality rate also increased, the bottom heatmap represents the change of 12 ferroptosis-related lncRNAs as the risk score increases; **C**. The AUC values of the patients' 1, 3, 5-year survival rate; **D**. the AUC of the signature and other traditional clinicopathological features.

**Table S1:** The detailed information of univariate COX analysis and multivariate COX analysis based on ferroptosis-related genes.

**Table S2:** The detailed information of univariate COX analysis and multivariate COX analysis based on ferroptosis-related lncRNAs.
